# Household food security and adequacy of child diet in the food insecure region north in Ghana

**DOI:** 10.1371/journal.pone.0177377

**Published:** 2017-05-11

**Authors:** Pascal Agbadi, Helga Bjørnøy Urke, Maurice B. Mittelmark

**Affiliations:** Department of Health Promotion and Development, Faculty of Psychology, University of Bergen, Bergen, Norway; Western Sydney University, AUSTRALIA

## Abstract

**Background and objectives:**

Adequate diet is of crucial importance for healthy child development. In food insecure areas of the world, the provision of adequate child diet is threatened in the many households that sometimes experience having no food at all to eat (household food insecurity). In the context of food insecure northern Ghana, this study investigated the relationship between level of household food security and achievement of recommended child diet as measured by WHO Infant and Young Child Feeding Indicators.

**Methods:**

Using data from households and 6–23 month old children in the 2012 *Feed the Future* baseline survey (n = 871), descriptive analyses assessed the prevalence of minimum meal frequency; minimum dietary diversity, and minimum acceptable diet. Logistic regression analysis was used to examine the association of minimum acceptable diet with household food security, while accounting for the effects of child sex and age, maternal -age, -dietary diversity, -literacy and -education, household size, region, and urban-rural setting. Household food security was assessed with the Household Hunger Scale developed by USAID’s Food and Nutrition Technical Assistance Project.

**Results:**

Forty-nine percent of children received minimum recommended meal frequency, 31% received minimum dietary diversity, and 17% of the children received minimum acceptable diet. Sixty-four percent of the children lived in food secure households, and they were significantly more likely than children in food insecure households to receive recommended minimum acceptable diet [O.R = 0.53; 95% CI: 0.35, 0.82]. However, in 80% of food secure households, children did not receive a minimal acceptable diet by WHO standards.

**Conclusions:**

Children living in food secure households were more likely than others to receive a minimum acceptable diet. Yet living in a food secure household was no guarantee of child dietary adequacy, since eight of 10 children in food secure households received less than a minimum acceptable diet. The results call for research into factors besides household food security in the search for determinants of child diet adequacy. In this study at least, household food security was a very weak marker of child diet adequacy. This finding is of significance to public health practice, since it calls into question any assumption that having enough food in a household necessarily results in adequately fed children.

## Introduction

The 1,000 days between conception and a child’s second birthday is a window of unsurpassed opportunity for child health promotion [1,2]. Exclusive breastfeeding up to six months and adequate complementary feeding up to two years of age fosters normal child growth and development, and is a foundation for health long after childhood [3–10]. Conversely, the consequences of inadequate complementary feeding practices may include illness, future learning disabilities, and inadequate future work capacity [1, 11].

Complementary feeding is “the process starting when breast milk is no longer sufficient to meet the nutritional requirements of infants, and therefore other foods and liquids are needed, along with breast milk” [11]. Adequate complementary feeding is defined by the World Health Organization in these terms [12]:

- A child is considered to receive at least the minimum dietary diversity (MDD) for health if breastfed and provided complementary food from four or more of seven food groups, or not breastfed and provided food from four or more of six food groups.
- A child is considered to receive at least the minimum meal frequency (MMF) for health if (i) at ages six to eight months she is breastfed and receives two or more daily feedings of solid, semi-solid or soft foods; or (ii) at ages nine to 23 months she is breastfed and receives three or more daily feedings of solid, semi-solid or soft foods; or (iii) at ages six to 23 months she is not breastfed and receives four or more daily feedings of solid, semi-solid or soft foods.
- A child is considered to receive at least the minimum acceptable diet (MAD) for health if the MDD and the MMF criteria are met.

Estimates from the period 2002 to 2008 show that globally, only a third of 6–23 months old infants and children received MDD, just half received MMF, and only a fifth received MAD [13]. This is an alarming shortfall from the ideal that all children have the need and right to sufficient food to support healthy development.

In 2008, only an estimated 47 percent of 6–23 months old infants and children received MDD, 50 percent received MMF, and 27 percent received MAD in Ghana [14]. The northern regions of Ghana are of particular concern due to higher than average levels of poverty, a relative lack of accessible health care, climate change challenges, and inadequate access to sufficient potable water and sanitation facilities. As of 2008, among 6–23 month old infants and children in Upper West, only 48 percent received MAD [15]. The corresponding findings for MAD in the other regions were 46 percent in Brong-Ahafo, 28 percent in Upper East and 22 percent in Northern region [15]. Thus, across the north, the majority of Ghanaian children do not receive adequate complementary feeding during the critical weaning period. The 2012 Feed the Future Population Based Survey (FTF-PBS) results indicated that only 16 percent of 6–23 months old infants and children received MAD in the northern regions of Ghana combined [16].

### Food security: Definition and measures

Food security is a complex and multidimensional concept; thus its definition varies among disciplines, institutions, and cultures [17]. A widely accepted definition developed by the Food and Agriculture Organization (FAO) states that food security occurs when “people at all times have physical, social and economic access to sufficient and nutritious food that meets their dietary needs for a healthy and active life”[18]. Food availability, economic and physical access to food, food utilization and stability are the four main dimensions captured in the definition of food security [19].

Considering the complexity of the food security concept, various measures have been developed as proxies of one or more dimensions of it. Food security is measured at the individual, household, community, and national levels. Some studies rely on an individual’s 24 hour recall of food consumption [20]. The US National Household Food Security Scale Module (HFSSM), the Household Food Insecurity Access Scale (HFIAS), the Household Hunger Scale (HHS), and the Household Consumption and Expenditure Surveys (HCES) are some examples of validated household level food security measures [21,22]. The global food security index is an example of a national or inter-country food security measure, and it considers affordability, availability, and quality and safety of food in the determination of the food security index scores (100 points score) [23].

Despite the plethora of food security measures in the literature, some commonalities were brought to light in a conceptual review of the existing understandings of food security across cultures. In this conceptual review, the authors argued that food insecurity measurements can only be considered complete when they consider, beyond food access and availability, food utilization and asset creation as significant indicators of food insecurity [17]. They further argued that food insecurity occurrences have socio-cultural and political underpinnings, and thus must be considered when designing food security measurements [17].

The 2016 global food security index (GFSI) indicates that food security globally is improving [23]. In sub-Saharan Africa, South Africa ranked first (and ranked 47^th^ among 113 countries globally) with a GFSI score of 62.9 [23]. Ghana ranked 3^rd^ out of 28 countries in sub Saharan Africa (and ranked 78^th^ among 113 countries globally) with a GFSI score of 47.8 [23].

### Household food security and child diet

There is some evidence from the Global South suggesting a positive relationship between household food security and practicing complementary feeding as recommended through the WHO IYCFI [24], child dietary diversity [25], and child dietary intake [26].

Plausible causes of food insecurity in developing countries include poverty, poor food policy environments, climate change and untoward climate events, inadequate food production, and criminality/corruption in the distribution of food [27,28]. Whatever the underlying causes, in Ghana’s north nearly four in ten households experience moderate to severe hunger [16,29]. In light of this, and the limited existing empirical research, it is plausible that a factor contributing to the low rates of MAD in northern Ghana is household food insecurity. Yet this possibility has not yet been investigated empirically in Ghana as far as a literature search reveals. Furthermore, a positive association between household food security and MAD is only one conceivable relationship. Also possible is a form of altruistic distribution of household foodstuffs in homes with poor food security, with adults sacrificing their own intake to ensure MAD. Equally possible is preferential food distribution, with privileged household members receiving food to the detriment of MAD. Thus, the relationship may actually be insignificant, positive, or negative. Factors other than household food security may influence MAD, such as child sex, number of small children in the household, cultural mores, conditions of urban/rural living, and so forth [30–35]. Any analysis of the relationship between household food security and MAD would need to consider such factors.

Following from the above, the relationship in the north of Ghana between household food security and MAD (and its components MDD and MMF) was investigated in this study. The following hypothesis was examined: Mothers from food insecure households in the north of Ghana are at higher risk of reporting inadequate diet provision to children 6–23 months compared with mothers from food secure households.

## Materials and methods

### Design

This study is a secondary data analysis of the Ghana 2012 Feed the Future Population Baseline Survey—FTF-PBS [36] (S1 Dataset). The FTF-PBS employed two-staged probability sampling in four regions north in Ghana. Firstly, 230 enumeration areas (EA) were selected by the Ghana Statistical Service (GSS) based on the 2010 Ghana Census Data. Secondly, 4600 households were selected from the 230 EAs by selecting 20 households from each EA [16].

### Study area

The study area is Brong Ahafo, Northern, Upper East, and Upper West regions of Ghana with an estimated population of 4.9 million. The predominant occupations are in agriculture. About 85 percent of adults have no formal education [16], and an estimated 22 percent of people in the study are classified as living in poverty [37]. Almost all the districts within the study area have social vulnerability to climate change [38]. By social vulnerability is meant individual’s or social grouping’s inability to adequately respond to, cope with, recover from or adapt to climate change [38]. Other social vulnerabilities in the study area include far distance from water sources, far distance from food market, female headed households, unimproved drinking water sources, high prevalence of malnourished children, illiteracy, agriculture employment, and lack of good road access, among others [38].

### Study sample

The FTF-PBS sampled 946 children from which data were collected from 871 children between 6–23 months old from 825 households, for a response rate of 92.1 percent [16]. Primary caregivers were interviewed to obtain data about the household and child: dwelling characteristics, household hunger experience, crop cultivation, food and non-food expenditure, women’s empowerment in agriculture, women’s dietary diversity, and breast- and complementary feeding (among other variables).

### Data collection

Data collection was done by trained enumerators from July through August, 2012 [16], using the Computer-Assisted Personal Interview (CAPI) method, although in some cases paper-based questionnaires were used [16]. The enumerators were unable to survey one EA due to flooding.

### Measures

#### Dependent variables

MDD, MMF and MAD were operationalised as per the WHO definitions already given in the Introduction. Children receiving the recommended feeding were coded 0 and all other children were coded 1. These are the food groups used in constructing the MDD and MAD, with data on feeding obtained through 24-hour recalls by mothers: grains, roots and tubers; legumes and nuts; dairy products (milk, yogurt, cheese); flesh foods (meat, fish, poultry and liver/organ meats); eggs; vitamin-A rich fruits and vegetables; and other fruits and vegetables [12].

#### Independent variables

Household food security was measured with the Household Hunger Scale (HHS). The HHS has been specifically developed as a meaningful measure of household food deprivation and has been validated for cross-cultural use [39]. The HHS is a revised 3 item 3 frequency (3I 3F) food insecurity scale from the 9 item 9 frequency (9I 9F) Household Food Insecurity Access Scale (HFIAS) [22]. The revision was arrived at after applying statistical methods based on the Rasch measurement model to assess the internal, external and cross-cultural validity of the HFIAS by using seven HFIAS datasets from Mozambique (two data sets), Malawi, West Bank/Gaza Strip, Kenya, Zimbabwe and South Africa [22]. Infit and outfit for most of the items of the HHS across the 7 datasets was within the range of 0.7–1.3, which is within the recommended range of a good fit [40].

The 3I 3F HHS is composed of the following items: no food of any kind to eat in the household because of lack of resources in the past month, a household member going to sleep at night hungry because there was insufficient food in the past month, and a household member going a whole day and night without eating anything at all because there was insufficient food in the past month. The response categories are never (0 times), rarely (1–2 times) or sometimes (3–10 times), and often (more than 10 times).

The HHS raw score is calculated for each household by summing the household’s responses to items 1, 2 and 3, where never = 0, rarely or sometimes1, and often2. The total raw score ranged between 0 and 6, and three categories were created from the raw score; that is, a score of 0–1 (little to no hunger), 2–3 (moderate hunger), and 4–6 (severe hunger).

Only five children were residents of severe hunger households, thus it was prudent to merge severe hunger households with moderate hunger households. Therefore, the HHS was used in this study to define two groups; households reporting (a) little to no hunger in the past month because of insufficient food or because of lack of resources to get food and thereby classified as food secure households, and (b) moderate to severe hunger in the past month because of insufficient food or because of lack of resources to get food, and thereby classified as food insecure households.

Cronbach alpha coefficients of the HHS have not previously been reported by the scale designers or other researchers. In the current study, the HHS demonstrated internal consistency (Cronbach’s alpha = 0.93); this was generated on the raw HHS. Collinearity seems within reason based on the mean inter-item correlation of 0.69. The high Cronbach alpha value suggest that one question (with its sub frequency occurrence question) in the scale may suffice in measuring hunger experiences of household members in the study area.

#### Extraneous (socio-demographic) variables

The socio-demographic (extraneous) variables used in this study were child sex, child age, maternal formal education (some education/no education), maternal English literacy (literate/illiterate), maternal dietary diversity, place of residence (urban/rural), region of residence (Brong Ahafo, Northern, Upper West, and Upper East), and household size. A large proportion of the women in the sample reported never having been to school (92.4%), and they were labelled *uneducated* while the remaining (7.6%), who reported some form of education ranging from primary to tertiary level were labelled *educated* [S1 Output]. Maternal English literacy was assessed by giving the respondents a text in English to read and a request to write. Those who could read and write in English were indicated as English literate. Maternal dietary diversity score was created using the 24-hour recall of mother’s consumption of foods from nine food groups: starchy staples (both cereal products and tubers, roots etc.); dark green leafy vegetables; other vitamin A rich fruits and vegetables; other fruits and vegetables; organ meat; Meat and fish; eggs; Legumes, nuts and seeds; and milk and milk products [41]. Maternal English literacy was assessed by giving the respondents a text in English to read and a request to write. Those who could read and write in English were indicated as English literate.

### Data analysis

IBM’s Statistical Package for Social Sciences (SPSS) version 22 was used to do the statistical analyses. Bivariate statistical analyses were carried out between the dependent and the independent variables. Outliers were also checked, and no outliers were observed. Finally, binary logistic regression modelling was performed for MAD.

### Informed consent

The enumerators were trained to obtain informed consent [16]. Respondents who could not express themselves in the English language were offered consent forms in their preferred language. Ethical clearance was obtained from the ethical review board of the University of Cape Coast [42]. The informed consent procedure was to explain the purpose of the study, and persons willing to participate gave consent by either signing a consent form or providing a thumbprint on the consent form. The signed consent forms were collected and filed at the METSS-Ghana office [16].

## Results

Patterns of breastfeeding and food intake by children are reported in Tables 1 and 2 respectively. A large majority of the children (93%) were breastfed a day or night before the survey. The majority of children were fed grains, roots, and tubers (81.5%) and vitamin-A rich fruits and vegetables (53.6%) the 24 hours before the survey.

**Table 1:** Breastfeeding patterns among 6–23 month old children in northern Ghana.

*Age group*	*Child ever been breastfed*		*Child breastfed day or night before the survey*	
	Breastfed (%)	Not breastfed (%)	Breastfed (%)	Not breastfed (%)
*6–11 months*	314 (99.7)	1 (0.30)	308 (97.8)	7 (2.20)
*12–17 months*	360 (98.6)	5 (1.40)	346 (94.8)	19 (5.20)
*18–23 months*	178 (93.2)	13 (6.80)	156 (81.7)	35 (18.3)
*6–23 months*	852 (97.8)	19 (2.20)	810 (93.0)	61 (7.00)

**Table 2:** Percentage of food intake among children according to food groups.

Food group	Food items	Number of children who received	
		Received (%)	Not received (%)
**Grains, Roots and Tubers**	Thin porridge, bread, rice, noodles, porridge or other foods made from grains (kenkey, banku, koko, tuo zaafi, akple), white potatoes, white yams, manioc, cassava, cocoyam, fufu or any other foods made from roots, tubers or plantain.	710 (81.5)	161 (18.5)
**Vitamin-A rich Fruits and Vegetables**	Pumpkin, red or yellow yams, carrots, sweet potatoes that are yellow or orange inside, any dark green, leafy vegetables (kontomire, aleefu, ayoyo, kale, cassava leaves), ripe mangoes, pawpaw, foods made with red palm oil, red palm nut, or red palm nut pulp sauce	467 (53.6)	404 (46.4)
**Flesh Foods**	Any meat, such as beef, pork, lamb, goat, chicken, or duck, fresh or dried fish or shellfish [e.g. prawn, lobster]	397 (45.6)	474 (54.4)
**Other Fruits and Vegetables**	Any other fruits or vegetables [e.g. bananas, avocados, tomatoes, oranges, apples]	232 (26.6)	639 (73.4)
**Dairy Products**	Infant formula such as winning mix or commercially produced infant formula, milk such as tinned, powdered, or fresh animal milk, yogurt, cheese, or other milk products	185 (21.2)	686 (78.8)
**Legumes and Nuts**	Any foods made from beans, peas, lentils, nuts, or seeds	162 (18.6)	709 (81.4)
**Eggs**	Eggs	84 (9.60)	787 (90.4)

The majority of women reported consuming meat and fish (78.6%), but smaller proportions reported consuming the other food groups (from 6%-50.4%) the 24 hours before the survey (Table 3).

**Table 3:** Percentage of food intake among mothers according to food groups.

Food groups	Food items	Proportion of mothers who ate from any of the food groups	
		Eaten (%)	Not Eaten (%)
Starchy staples (both cereal products and tubers, roots etc.)	Bread, rice, noodles, or other foods made from grains (kenkey, banku, koko, tuo zaafi, akple, weanimix), white potatoes, white yams, manioc, cassava, cocoyam, fufu or any other foods made from roots, tubers or plantain,	107 (12.3)	764 (87.7)
Dark green leafy vegetables	Pumpkin, red or yellow yams, carrots, sweet potatoes that are yellow or orange inside, Any dark green, leafy vegetables (kontomire, aleefu, ayoyo, kale, cassava leaves)	237 (27.2)	634 (72.8)
Other vitamin A rich fruits and vegetables	Any other fruits or vegetables [e.g. bananas, avocados, tomatoes, oranges, apples], foods made with red palm oil, red palm nut, or red palm nut pulp sauce	439 (50.4)	432 (49.6)
Other fruits and vegetables	Ripe mangoes, pawpaw	52 (6.0)	819 (94.0)
Organ meat	Liver, kidney, heart or other organ meats	54 (6.2)	817 (93.8)
Meat and fish	Any meat, such as beef, pork, lamb, goat, chicken, or duck, fresh or dried fish or shellfish [e.g. prawn, lobster]	685 (78.6)	186 (21.4)
Eggs	Eggs	89 (10.2)	782 (89.8)
Legumes, nuts and seeds	Any foods made from beans, peas, lentils, nuts, or seeds	297 (34.1)	574 (65.9)
Milk and milk products	Milk such as tinned, powdered, or fresh animal milk, yogurt, cheese, or other milk products	177 (20.3)	694 (79.7)

Descriptive statistics for all study variables are shown in Table 4. Four hundred and twenty-three (49.1%), 268 (30.8%), and 145 (16.6%) of children reportedly received the recommended MMF, MDD, and MAD, respectively. Three-hundred and fourteen (36.2%) children lived in food insecure households.

**Table 4:** Descriptive statistics of dependent, main independent, and extraneous (socio-demographic) variables.

	N (%)	Mean	SD
***Dependent variables***			
**Minimum Meal Frequency**			
**Received**	423 (49.1)		
Did not receive	438 (50.9)		
**Missing**	10		
**Minimum Dietary Diversity**			
**Received**	268 (30.8)		
Did not receive	603 (69.2)		
**Minimum Acceptable Diet**			
**Received**	145 (16.6)		
Did not receive	726 (83.4)		
***Main food security variable***			
**Household Hunger Scale**			
**Little to no hunger (food secure)**	555 (63.9)		
**Moderate hunger (food insecure)**	309 (35.6)		
**Severe hunger (food insecure)**	5 (0.6)		
**Missing**	2		
**Maternal education**			
**Educated**	66 (7.6)		
**Uneducated**	805 (92.4)		
**English literacy**			
**Literate**	93 (89.1)		
**Illiterate**	763 (10.9)		
**Missing**	15		
**Age of mother/guardian**	861 (100)	29.04	7.484
**Region**			
**Brong Ahafo**	104 (11.9)		
**Northern**	572 (65.7)		
**Upper East**	120 (13.8)		
**Upper West**	75 (8.6)		
**Place of residence**			
**Rural**	725 (83.2)		
**Urban**	146 (16.8)		
**Household size**	871 (100)	7.20	3.873
**Sex of Child**			
**Girls**	430 (49.4)		
**Boys**	441 (50.6)		
**Age of Child**			
**6–11months**	315 (36.2)		
**12–17months**	365 (41.9)		
**18–23months**	191 (21.9)		

Chi-square tests of independence showed that level of household food security and child age were significantly associated with MDD and MAD but not with MMF (Table 5).

**Table 5:** Results of chi-square test and descriptive statistics for minimum meal frequency, minimum dietary diversity, and minimum acceptable diet, and categorical variables.

Variables	Minimum Meal Frequency				Minimum Dietary Diversity				Minimum Acceptable Diet			
	No	Yes	χ^2^	df	No	Yes	χ^2^	df	No	Yes	χ^2^	df
**Child sex**												
Female	216 (49.3%)	207 (48.9%)	0.00^a^	1	285 (47.3%)	145 (54.1%)	3.21^a^	1	348 (47.9%)	82 (56.6%)	3.25^a^	1
Male	222 (50.7%)	216 (51.1%)			318 (52.7%)	123 (45.9%)			378 (52.1%)	63 (43.4%)		
**Child Age**												
6–11 months	164 (37.4%)	151 (35.7%)	0.51	2	263 (43.6%)	52 (19.4%)	60.35*	2	286 (39.4%)	29 (20.0%)	19.89*	2
12–17 months	177 (40.4%)	181 (42.8%)			242 (40.1%)	123 (45.9%)			287 (39.5%)	78 (53.8%)		
18–23 months	97 (22.1%)	91 (21.5%)			98 (16.3%)	93 (34.7%)			153 (21.1%)	38 (26.2%)		
**Household hunger scale**												
Food secure household	268 (61.5%)	277 (65.5%)	1.33^a^	1	362 (60.1%)	193 (72.3%)	11.32^a^**	2	446 (61.6%)	109 (75.2%)	9.06**	2
Food insecure household	168 (38.5%)	146 (34.5%)			240 (39.9%)	74 (27.7%)			278 (38.4%)	36 (24.8%)		

Numbers in parentheses indicate column percentages.

^a^ = Yates’ Correction of Continuity.

*p < .0001,

**p<0.005

### Logistic regression analysis

The results for MAD regressed on all other variables are shown in Table 6. Accounting for extraneous (socio-demographic) variables, household food security (as measured by the Household Hunger Scale) was significantly associated with MAD. The variance in MAD accounted for (r^2^) by the logistic regression model was estimated between 0.09 and 0.14. Maternal dietary diversity was also a significant correlate of MAD. Child age was a significant correlate of MAD, with children in the two oldest age groups more likely to receive MAD than 6–11 months old children.

**Table 6:** Minimum acceptable diet received by children regressed on household food security variables, maternal resources and contextual variables.

Variables	B	S.E.	Wald	df	Sig.	O.R.	95% C.I. for O.R.	
							Lower	Upper
Household Food Security (reference: food insecure)	-0.55	0.22	5.97	1	0.02	0.58	0.37	0.90
Maternal dietary diversity (continuous)	-0.37	0.07	30.28	1	0.00	0.69	0.60	0.79
Maternal Education (reference: uneducated)	-0.17	0.48	0.12	1	0.73	0.84	0.33	2.18
Maternal English Literacy (reference: illiterate)	-0.17	0.43	0.16	1	0.69	0.84	0.36	1.97
Maternal Age (continuous)	-0.02	0.01	1.58	1	0.21	0.98	0.96	1.01
Sex of Child (reference: boy)	-0.39	0.20	3.89	1	0.05	0.68	0.46	1.00
Age of Child (reference: 6–11 months)			19.28	2	0.00			
12–17 months	-0.86	0.28	9.73	1	0.00	0.42	0.25	0.73
18–23 months	-1.06	0.24	18.89	1	0.00	0.35	0.21	0.56
Locality (reference: rural)	0.12	0.27	0.16	1	0.69	1.11	0.66	1.89
Brong Ahafo (reference)			6.81	3	0.08			
Northern	0.16	0.31	0.27	1	0.60	1.17	0.65	2.13
Upper East	0.58	0.41	2.03	1	0.15	1.79	0.80	3.98
Upper West	-0.50	0.42	1.47	1	0.23	0.60	0.27	1.37
Household size (continuous)	0.02	0.03	0.66	1	0.42	1.02	0.97	1.08
Constant	4.62	0.64	52.05	1	0.00	101.04		

Overall model fit estimates: R square range: 0.09–0.14. χ^2^ = 74.99. degrees of freedom = 13. O.R. indicates odds ratio. C.I. indicates confidence intervals

Considering the significant contribution of child age in the logit model in Table 6, another logit regression (in Table 7) was performed to control for child age as a continuous function of age in months and months squared because age is expected to show a non-linear relationship to minimum acceptable diet. Although the effect was small, feeding adequacy improved with age.

**Table 7:** Minimum acceptable diet received by children regressed on household food security variables, maternal resources, and contextual variables, with age of child in month squared.

	B	S.E.	Wald	df	Sig.	O.R.	95% C.I. for O.R.	
							Lower	Upper
Household Food Security (reference: food insecure)	-0.51	0.22	5.24	1.00	0.02	0.60	0.39	0.93
Maternal dietary diversity (continuous)	-0.38	0.07	30.29	1.00	0.00	0.69	0.60	0.79
Maternal Education (reference: uneducated)	-0.11	0.49	0.05	1.00	0.82	0.89	0.34	2.31
Maternal English Literacy (reference: illiterate)	-0.17	0.43	0.16	1.00	0.69	0.84	0.36	1.96
Maternal Age	-0.02	0.01	1.57	1.00	0.21	0.98	0.96	1.01
Age of child in month	-0.35	0.13	6.76	1.00	0.01	0.70	0.54	0.92
Age of child in month squared	0.01	0.00	4.19	1.00	0.04	1.01	1.00	1.02
Sex of Child (reference: boy)	-0.37	0.20	3.48	1.00	0.06	0.69	0.47	1.02
Locality (reference: rural)	0.12	0.27	0.18	1.00	0.67	1.12	0.66	1.90
Brong Ahafo (reference)			6.46	3.00	0.09			
Northern	0.09	0.30	0.08	1.00	0.77	1.09	0.60	1.98
Upper East	0.54	0.41	1.75	1.00	0.19	1.72	0.77	3.84
Upper West	-0.53	0.41	1.67	1.00	0.20	0.59	0.26	1.32
Household size (continuous)	0.02	0.03	0.59	1.00	0.44	1.02	0.97	1.08
Constant	6.81	1.13	36.36	1.00	0.00	905.00		

Overall model fit estimates: R square range: 0.08–0.14. χ^2^ = 74.99. degrees of freedom = 13. O.R. indicates odds ratio. C.I. indicates confidence intervals

Analyses stratified by child sex were conducted to ascertain the contribution of food security, maternal diet and socio-demographic variables to boys and girls (Tables 8 and 9). Maternal dietary diversity and child age were statistically significant among both boys and girls in northern Ghana. In addition, compared to boys in Brong Ahafo region, boys in Upper West region were less likely to be inadequately fed (Table 9).

**Table 8:** Minimum acceptable diet received by children regressed on household food security variables, maternal resources and contextual variables for girls.

	B	S.E.	Wald	df	Sig.	O.R.	95% C.I. for O.R.	
							Lower	Upper
Household Food Security (reference: food insecure)	-0.52	0.31	2.80	1	0.09	0.60	0.32	1.09
Maternal dietary diversity (continuous)	-0.39	0.09	17.27	1	0.00	0.68	0.56	0.81
Maternal Education (reference: uneducated)	0.22	0.68	0.11	1	0.74	1.25	0.33	4.72
Maternal English Literacy (reference: illiterate)	-0.75	0.61	1.50	1	0.22	0.47	0.14	1.57
Maternal Age	-0.01	0.02	0.31	1	0.58	0.99	0.95	1.03
Age of Child (reference: 6–11 months)			9.78	2	0.01			
12–17 months	-1.03	0.37	8.04	1	0.01	0.36	0.17	0.73
18–23 months	-0.94	0.34	7.73	1	0.01	0.39	0.20	0.76
Locality (reference: rural)	-0.26	0.36	0.53	1	0.47	0.77	0.38	1.57
Brong Ahafo (reference)			1.95	3	0.58			
Northern	0.25	0.41	0.36	1	0.55	1.28	0.57	2.86
Upper East	0.72	0.53	1.84	1	0.18	2.05	0.73	5.75
Upper West	0.25	0.64	0.15	1	0.70	1.28	0.37	4.45
Household size (continuous)	0.04	0.04	0.95	1	0.33	1.04	0.96	1.13
Constant	3.89	0.85	21.02	1	0.00	49.03		

Overall model fit estimates: R square range: 0.10–0.16. χ^2^ = 42.46. Degrees of freedom = 12. O.R. indicates odds ratio. C.I. indicates confidence intervals

**Table 9:** Minimum acceptable diet received by children regressed on household food security variables, maternal resources and contextual variables for boys.

	B	S.E.	Wald	df	Sig.	O.R.	95% C.I. for O.R.	
							Lower	Upper
Household Food Security (reference: food insecure)	-0.61	0.34	3.24	1	0.07	0.54	0.28	1.06
Maternal dietary diversity (continuous)	-0.32	0.10	9.97	1	0.00	0.73	0.60	0.89
Maternal Education (reference: uneducated)	-0.73	0.78	0.86	1	0.35	0.48	0.10	2.24
Maternal English Literacy (reference: illiterate)	0.39	0.70	0.31	1	0.58	1.47	0.38	5.76
Maternal Age	-0.04	0.02	3.71	1	0.05	0.96	0.92	1.00
Age of Child (reference: 6–11 months)			11.87	2	0.00			
12–17 months	-0.61	0.45	1.84	1	0.18	0.55	0.23	1.31
18–23 months	-1.24	0.37	11.41	1	0.00	0.29	0.14	0.59
Locality (reference: rural)	0.59	0.43	1.85	1	0.17	1.80	0.77	4.19
Brong Ahafo (reference)			10.20	3	0.02			
Northern	0.04	0.48	0.01	1	0.94	1.04	0.41	2.64
Upper East	0.65	0.69	0.87	1	0.35	1.91	0.49	7.43
Upper West	-1.22	0.60	4.10	1	0.04	0.29	0.09	0.96
Household size (continuous)	0.03	0.04	0.51	1	0.47	1.03	0.96	1.10
Constant	5.24	.099	27.90	1	0.00	188.59		

Overall model fit estimates: R square range: 0.09–0.17. χ^2^ = 41.85. Degrees of freedom = 12. O.R. indicates odds ratio. C.I. indicates confidence intervals

## Discussion

### Household food security

This study observed that household food security was a significant correlate of child MAD, suggesting that food secure households may have access to resources that enabled them to overcome frequent, widespread food insecurity that is prevalent in the northern regions of Ghana. These findings are in line with previous research by Osei et al finding significant differences in MMF, MDD and MAD between food secure and food insecure households favouring food secure households. Similarly, Humphries et al [25] found child dietary diversity to mediate the relationship between household food security and child anthropometry in children of 5 years, and hence indicating a relationship between household food security and one aspect of child diet.

Bivariate results in this study showed that household food security was significantly associated with MDD, but not with MMF. This is an indication that the lower likelihood of MAD in food insecure households as shown in regression analyses, is mainly due to failure in meeting the MDD. This indicates that in the context of northern Ghana, children in food insecure households in this study received enough, but not adequately diverse food. Knowing that chronic malnutrition is largely due to lack of a diverse diet (including proteins and micronutrients), and the serious short- and long term consequences of chronic malnutrition, the findings of this study underscore the urgency of improving child diet in northern Ghana.

Of further interest in this study, therefore, is the question of what factors lead to household food security in this vulnerable area. Agriculture production and household food security are related [43–45], so it may be that food secure households in the study area had access to farmland, and thereby stored foodstuffs available for consumption even in food- insecure periods in the region. This could not be investigated with the present data due to lack of information about household access to agricultural land. Yet the relationship between household food security and child MAD was far from unitary: even in the food secure households, the majority of children did not receive a MAD. This is a caution against the notion that simply increasing the proportion of food secure households will result in significant improvement in child diet. There were instances of children receiving a MAD while living in food insecure households. Therefore, household feeding priorities [46–48] and altruistic behaviour or food buffering of children regardless of household food security-insecurity status may be an important moderator of the link between household food security and child diet. However, this possibility could not be investigated with the present data due to lack of data on household feeding priorities, altruistic behaviour or food buffering of children.

### Maternal dietary diversity

Maternal dietary diversity was a significant correlate in both the full sample and the sex-stratified analyses. Mothers whose diets were diverse were more likely to give their children adequate complementary feeding. The protective effect of maternal dietary diversity against inadequate MAD in northern Ghana is consistent with findings of studies in sub Saharan Africa and South and East Asia and Latin America [35,49,50].

### Child age

The results from this study indicated that child complementary feeding improved with age, with older children being more likely to receive MAD. This result confirms findings from similar studies in sub-Saharan and South and East Asian countries that observed that children between 12–23 months were more likely to achieve MAD [33,34,51,52]. However, in Ghana and Nigeria, 6–11 months old children are more likely to receive MAD [32]. The Ghana 2008 DHS data was nationally representative, whereas in the present study the sample focused on regions in the northern part of Ghana. This difference in representativeness may be the reason for the contradiction in the results of this study and that of the nationally representative study of Ghana [32].

### Socio-demographic (extraneous) variables

Maternal education and literacy, region of residence and place of residence (rural/urban) were not statistically significantly associated with MAD in the full sample. One possible explanation of these results is that the majority of children in our study sample lived with illiterate and uneducated mothers, lived in northern region, and lived in rural areas. Other ways of measuring maternal knowledge, like informal knowledge or beliefs about health, diet and childcare might prove more useful in resource poor settings like that of this study.

### Policy implications

Various nutrition research and policy intervention models hypothesise that household food security is a significant determinant of infant and young child feeding practices and nutritional outcome [47,48]. However, in line with the result of the present analyses, it is evident that advocating for policies that seek to promote household food security as a pathway to achieving child feeding adequacy may have minimal impact, at least in Ghana, and possibly in many other low and middle income countries.

The results of this study also indicate that children within the youngest age group were at risk of being underfed, but the fact that there were cases of inadequate acceptable diet in the older age group implies that health promotion interventions and policies should adopt an all-inclusive approach in addressing child malnutrition.

### Study limitations

This study used data from a cross-sectional survey; therefore, the analyses cannot establish causal relationships. The conclusions in this study are restricted to associations between the independent and the dependent variables. There are two methodological challenges related to the HHS. The four-week recall method relies heavily on the memory of informants. A household head may not have adequate knowledge about food insecurity occurrences of each member of the household; each individual in the household may have differing opinions on hunger experiences. In addition, the FTF-PBS used only HHS as a household food security measure. Thus, it was impossible in this study to ascertain how HHS relates with other food security measures. However, a study conducted in Sidama District in Ethiopia found a Pearson correlation coefficient of 0.67 between HFIAS and HHS, suggesting that HHS is a promising measure of food security.

Another limitation relates to the extremely skewed distribution of the maternal education variable in the FTF-PBS dataset. A very small proportion of women in the sample reported having any level of formal education, be it primary, secondary, or tertiary education. These were therefore combined in one category and the education variable was coded ‘no education’ versus ‘some education’. It was therefore not feasible to examine the present data for a possible relationship between different levels of maternal education and child diet. Such a relationship might be observed in other samples with less skewed data.

### Conclusions

The objective of this study was to investigate the relationship between household food security and child feeding adequacy, and the results show a significant statistical association after accounting for child sex and age and other sociodemographic variables. Importantly, while household food security was related to child feeding adequacy, there were large numbers of underfed children in food secure households and of well-fed children in food insecure households in this study. Furthermore, the logistic regression analysis revealed that household food security accounted for less than 10% of the variance in child feeding adequacy. We propose that from a public health standpoint, household food security is a poor marker/indicator of child feeding adequacy, but this calls for confirmatory research.

## Supporting information

S1 Dataset Data underlying the results of the study. Data file (in SPSS).
(SAV)

S1 Output Output for education variable. Output file (in PDF).
(PDF)
