# Supplementary material for: Household food security and adequacy of child diet in the food insecure region north in Ghana
Source: PLoS One. 2017 May 11;12(5):e0177377. doi: 10.1371/journal.pone.0177377 (PMC5426760; doi:10.1371/journal.pone.0177377)
Supplement: S1 Output — Output file (in PDF). (PDF) [file pone.0177377.s002.pdf]

```
FREQUENCIES VARIABLES=education_level q2_07
  /ORDER=ANALYSIS.
```

Frequencies

Notes

Output Created		02-APR-2017 09:31:46
Comments		
Input	Data	C:\Users\Eyram-Eyram\Desktop\Okay\330 VariablesIndividualLevelAnalysisSTRATIFIED---.sav
	Active Dataset	DataSet3
	Filter	<none>
	Weight	<none>
	Split File	<none>
	N of Rows in Working Data File	871
Missing Value Handling	Definition of Missing	User-defined missing values are treated as missing.
	Cases Used	Statistics are based on all cases with valid data.
Syntax		FREQUENCIES VARIABLES=education_level q2_07 /ORDER=ANALYSIS.
Resources	Processor Time	00:00:00.03
	Elapsed Time	00:00:00.04

```
[DataSet3] C:\Users\Eyram-Eyram\Desktop\Okay\330 VariablesIndividualLevelAnalysisSTRATIFIED---.sav
```

Statistics

		education_level Highest Education Level	q2_07 Can and write English (English Literacy)
N	Valid	871	856
	Missing	0	15

Frequency Table

education_level Highest Education Level

		Frequency	Percent	Valid Percent	Cumulative Percent
Valid	0 None	805	92.4	92.4	92.4
	1 MSLC	1	.1	.1	92.5
	2 BECE	37	4.2	4.2	96.8
	4 Teacher Train A	2	.2	.2	97.0
	7 SSCE/WASSCE	21	2.4	2.4	99.4
	10 Tech/Prof Dip	2	.2	.2	99.7
	11 HND	1	.1	.1	99.8
	15 Other	2	.2	.2	100.0
	Total	871	100.0	100.0	

q2_07 Can and write English (English Literacy)

		Frequency	Percent	Valid Percent	Cumulative Percent
Valid	0 Cannot Read and Write English	763	87.6	89.1	89.1
	1 Can write English Only	2	.2	.2	89.4
	2 Can Read English only	9	1.0	1.1	90.4
	3 Can read and write English	82	9.4	9.6	100.0
	Total	856	98.3	100.0	
Missing	System	15	1.7		
Total		871	100.0		
